# Genetic insights into Tetralogy of Fallot: Oh *MYH*(6)

**DOI:** 10.1038/s41390-024-03195-3

**Published:** 2024-04-10

**Authors:** Rahul Mital, John S. Lozier, Timothy J. Mead

**Affiliations:** 1 Division of Pediatric Cardiology, University Hospitals Rainbow Babies and Children’s Hospital, Cleveland, OH USA; 2 Department of Pediatrics, Case Western Reserve University School of Medicine, Cleveland, OH USA

Congenital heart defects (CHDs) are the most common birth defect with an incidence of approximately 7–9 per 1000 live births worldwide.^1^ Of these, Tetralogy of Fallot (ToF) is the result of an anterior and cephalad deviation of the infundibular septum during cardiogenesis, resulting in ventricular septal defect (VSD; lack of septation of the left and right ventricle); overriding aorta (malalignment of the aorta over the intraventricular septum); pulmonary stenosis (PS; narrowing of the right ventricular outflow tract to the pulmonary arteries), and right ventricular hypertrophy (RVH; thickening of the right ventricular wall secondary to right ventricular hypertension). ToF occurs in approximately 3–5 out of 10,000 live births, and accounts for 7–10% of CHDs.^2^ This defect, whether genetic or idiopathic in nature, results in cyanosis, and confers an increased risk of endocarditis, arrhythmia, fainting, seizures, stunted growth, and death.^3^ ToF is the most common cyanotic CHD of those surviving beyond the neonatal period, and is typically repaired within the first year of life.^4^ Despite its clinical significance, the genetic mechanisms underlying ToF are not completely clear.

In this issue of *Pediatric Research*, Zuo et al. explores the role of the gene myosin heavy chain 6 (*MYH6*) in ToF, a previously unidentified association.^5^ *MYH6* is a gene that encodes for cardiac alpha myosin heavy chain, a component of type II myosin, a protein integral for cardiac contraction. *MYH6* mutations, specifically those interfering with myosin heavy chain and light chain interactions, have been associated with dilated cardiomyopathy (DCM), and hypertrophic cardiomyopathy (HCM).^6^ Most commonly however, mutations in *MYH6* have been associated with atrial septal defects (ASDs), with previous studies showing *MYH6* knock-down chick embryos with incomplete atrial septation.^7^ In a previously published study, Zuo et al. demonstrated that variants in the *MYH6* gene promotor region may possibly affect myocardial development and lead to VSD formation.^8^ The authors hypothesized that *MYH6* may also play a role in the pathogenesis of ToF, given previous associations noted between this particular CHD and mutations in myogenic heart progenitors.

Through a series of elegant experiments completed in the context of a large retrospective study consisting of 608 participants including subjects both with and without ToF, Zuo et al. identified 12 total variants in the *MYH6* gene promoter region, and focused specifically on the five that were found only in ToF patients (two of which were novel mutations), and their effects on transcription factor binding sites (TFBS).^5^ Electrophoretic mobility shift assays showed that these variants modified TFBS on the *MYH6* promoter, likely altering function at the cellular level. Using a dual reporter luciferase assay, the authors interrogated the gene regulatory effect of these variants as compared to the wild type *MYH6* sequenced gene promoters. Ultimately, four of the variants were found to be transcriptionally significant, with a significant decrease in expression of the *MYH6* gene. Using the JASPAR transcription factor binding profiles database, the authors concluded that the transcription factors bound by the variants were altered, and many of the newly created TFBS may be implicated in the suppression of normal cardiovascular development. This study opens a new research focus area on the *MYH6* promoter region and makes a case to add *MYH6* as a causative ToF gene for genetic screening.

ToF presents an interesting paradox: As the most common cyanotic heart defect, and one that often requires both timely intervention and lifelong follow-up, the clinical understanding and management of ToF has continued to improve. However, a complete understanding of the genetic mechanisms underlying ToF remains elusive, with approximately 80% of cases being non-syndromic and without a known etiology.^9^ Pathological variants in cardiac transcription factors (*GATA4*, *GATA5*, *GATA6*) and certain transcription factors of the second heart field (*HAND2*, *FOXC1*, *FOXC2*, *FOXH1*, and *TBX1*) are frequently found in non-syndromic ToF patients.^10^ Despite these known associations, however, emerging candidate genes continue to be identified. For example, loss of function in *KDR*, *IQGAP1*, and *GDF1* genes have made them emerging candidate genes in ToF and CHD. Recent studies have indicated that deleterious variants in the *NOTCH1* and *FLT4* genes have significant implications in ToF and CHD, although the understanding of the mechanisms connecting these genes to CHD remains incomplete.^9,10^ Other studies have shown that patients with rare mutations in multiple different genes may develop CHDs as a result of the synergistic nature of these disturbances.^11^ The dynamic and ongoing nature of research surrounding the molecular basis of ToF only serves to highlight the complexity of the dysregulated genetic network implicated in the disease, and the need for continued investigation into the field.

The study from Zuo et al. is consequential for multiple reasons.^5^ Foremost, the study uncovers a potential new candidate gene that may be implicated in the pathophysiology of ToF. Previous mention of the role of *MYH6* has been anecdotal at best; a 2019 study by Xia et al. exploring the association between *MYH6* gene mutations and ASDs noted than an affected individual was not only diagnosed with an ASD, but ToF as well.^12^ In another study, mutations in Myomesin 2 (*MYOM2*) were identified in patients with ToF; when further analyzed in a drosophila animal model, the authors noted that compound heterozygous combinations of the *CG14964* gene (a *MYOM*2 analog) and *Mhc* (a *MYH6/7* analog), exhibited synergistic genetic interactions, resulting in multiple different abnormal cardiac phenotypes.^13^

The study by Zuo et al. is the first that focuses specifically on the role of *MYH6* in the pathogenesis of ToF.^5^ Follow-up of these findings with screenings for these mutations in a large cohort is warranted. In addition, creation of genetic mouse models, via CRISPR-Cas9 and base editing, to generate relevant SNPs to study the function of *MYH6* promoter may allow for further characterization of mutations of interest.

Through their work, Zuo et al. do a masterful job reinforcing the idea that there is a need for advanced sequencing to uncover novel genes and promoter regions of genes associated with CHD, including ToF.^5^ By continuing to uncover candidate genes, missing links in what is likely a complex multifactorial pathway with multiple gene-gene interactions underlying ToF can continue to be elucidated and, ultimately, lead to new therapeutic interventions in the clinical setting.
